# Severe Aortic Stenosis and Severe Coarctation of the Aorta: A Hybrid Approach to Treatment

**DOI:** 10.3389/fsurg.2017.00016

**Published:** 2017-03-17

**Authors:** Daniel McLennan, Massimo Caputo, Demetris Taliotis

**Affiliations:** ^1^Department of Cardiology, Bristol Royal Hospital for Children, University of Bristol, Bristol, UK; ^2^Department of Cardiac Surgery, Bristol Heart Institute, University of Bristol, Bristol, UK

**Keywords:** aortic stenosis, coarctation of the aorta, hybrid surgery, congenital heart disease, neonate

## Abstract

Hybrid surgery is becoming more popular in the treatment of children with congenital heart disease, particularly small infants and neonates. We report a case of a patient with aortic stenosis (AS) and coarctation of the aorta (CoA). Case: a 1-month-old baby presented with severe AS and CoA. The decision was made to perform a hybrid surgical procedure. The patient underwent a lateral thoracotomy for repair of the CoA and carotid cutdown for aortic balloon valvuloplasty (AoVP).

## Introduction

In the last century, management of congenital heart disease (CHD) has significantly changed. When the first pediatric cardiac surgery was performed in 1944 by Dr. Alfred Blalock, the goal was to stop “blue babies dying” (1). With advances in both the surgical and medical approach to CHD and the development of open-heart surgery and cardiopulmonary bypass, there are now surgical options for cardiac conditions which were previously inoperable. With these advances, the current aim is not just to reduce mortality in children with CHD but to reduce morbidity and improve quality of life.

The advent of hybrid surgical options for treating congenital heart problems has transformed the approach to palliating and repairing complex congenital cardiac conditions. Particularly in neonates and infants under 5 kg, it allows for the combined strengths of cardiologists and surgeons to perform procedures that will provide the best outcomes for patients with the hypothesized benefits of lower morbidity and mortality.

At Bristol Children’s Hospital in conjunction with the Bristol Heart Institute, there has been an increase in hybrid procedures being performed on both adults and children with CHD. A unique case where a hybrid approach was taken will be discussed.

## Case

The case is of a 3.7-kg, 1-month-old male patient who presented at day 24 of life with severe aortic stenosis (AS) and coarctation of the aorta (CoA). He was born at term following an uncomplicated pregnancy. On day 5 of life, he was noted to be jaundiced and have a murmur. His jaundice resolved, and he was discharged home with cardiac follow-up. He then had progressive increase in his work of breathing and was not gaining weight. He had an echocardiogram on day 24 of life which showed a thickened and dysplastic bicuspid aortic valve with doming of the valve leaflets. The flow across the valve was measured on continuous wave doppler at 4 m/s. He was also found to have a discrete juxta ductal coarctation of the aorta with continuous doppler flow of 2.5 m/s with diastolic tail. His ventricular function remained preserved. He was started on intravenous prostaglandin and urgent surgery was scheduled.

Due to the complexity of the case and the need for both aortic valve and aortic arch intervention, it was felt the best course of action would be a hybrid approach with surgical end-to-end anastomosis of the aortic arch *via* a thoracotomy followed by surgical carotid cut down for AoVP. This plan was made to avoid the need for a sternotomy on bypass to alleviate the aortic valve stenosis. The patient was taken to the hybrid catheter laboratory theater, where he was placed in the left lateral position, prepped and draped. He underwent the surgical end-to-end anastomosis *via* the thoracotomy *via* the third intercostal space, as planned. The arch, patent ductus arteriosus (PDA) and descending aorta were then dissected out. Clamps were applied to the arch and descending aorta. The coarctation segment was resected and PDA ligated. After this, an end to side anastomosis was performed between the aortic arch and the descending aorta. The pleura and chest were closed in layers.

At this point the patient was assessed and felt to be stable to proceed with the planned aortic balloon. The patient was turned from the lateral position to his back and the surgeon performed a carotid cutdown on the right common carotid artery. A 5/0 purse string suture was applied to the vessel, and through that a 4-Fr sheath was inserted in the right common carotid artery. Using a 4-Fr pigtail catheter, ascending aortic pressure was measured at 61/42 mmHg. An aortic angiogram was then performed and the aortic valve annulus measured 7.5 mm. The pigtail catheter was then replaced with a 4-Fr Judkins Right Coronary Catheter (JR). Using a Terumo 0.035″ guide wire the JR catheter was placed into the left ventricle (LV). The pressure in the LV was recorded at 110/13 mmHg, giving a peak to peak gradient of 49 mmHg. A 0.014 ChoICE PT extra support coronary wire (Boston Scientific) was then placed in the LV and the JR catheter removed. Next a NuMED Tyshak II 6 mm × 2 cm percutaneous transluminal valvuloplasty balloon catheter was chosen and delivered over the ChoICE wire to the aortic valve. The balloon was inflated to burst pressure of 4 atmospheres (atm) with waist seen within the balloon and dewaisting occurred. The balloon catheter was removed and the JR catheter re-introduced. The pressure was measured at 100/10 mmHg. Repeat angiogram at the aortic root at this stage did not demonstrate any significant aortic incompetence. The JR catheter and the Terumo wire were again used to recross the valve, and a 7 mm × 2 cm NuMED Tyshak II percutaneous transluminal valvuloplasty balloon catheter was chosen and delivered over the coronary guidewire in position. The balloon was inflated to 4 atm, a waist was again seen and dewaisting occurred. The balloon catheter was removed, and the JR catheter placed back into the LV. Pressure within the LV was now 90/10 mmHg with pullback peak to peak gradient between the LV and ascending aorta measuring 30 mmHg. The catheter and sheath were removed, the vessel was repaired by tightening the pursestring suture and the skin closed in layers.

An echocardiogram performed post procedure showed qualitatively normal ventricular function with no pericardial effusion. The arch was unobstructed with doppler flow velocity of 1.6 m/s. The aortic valve was bicuspid and obviously dysplastic. There was mild flow acceleration across the valve using continuous wave doppler of 3 m/s with mean doppler gradient of 15 mmHg. There was no obvious aortic incompetency seen.

The patient was transferred from the hybrid catheter laboratory to the pediatric intensive care unit (PICU). He remained cardiovascularly stable during his PICU admission. He was extubated but had to be reintubated within a few hours due to upper airway issues which prolonged his PICU stay. He was successfully extubated on day 3 post procedure and was transferred to the cardiac ward. His admission on the ward was uncomplicated and he was discharged on day 6 post intervention.

At 4 months of age, he was reviewed in the outpatient clinic. He was clinically asymptomatic with steady growth. His echocardiogram demonstrated qualitatively normal ventricular function, the aortic valve stenosis was mild to moderate with flow of 3.7 m/s on continuous wave doppler with mean gradient of 30 mmHg. There was evidence of mild aortic incompetence, his aortic arch remained unobstructed with flow velocity of 1.4 m/s. His most recent follow-up was at 8 months of age. He remained asymptomatic and well with his weight increasing to 9 kg. His echocardiogram again demonstrated qualitatively normal ventricular function, bicuspid aortic valve with flow of 3.2 m/s across the valve. There was mild aortic incompetence and the aortic arch remained unobstructed. There were no issues with his carotid artery access and there were no ongoing neurological issues.

## Discussion

Left ventricular outflow tract (LVOT) obstructive lesions account for approximately 4–6% of CHD with incidence of 4 per 10,000 live births (2). AS is the most common LVOT lesion accounting for 70%, with CoA being the second most common one (2). The combination of both severe AS and CoA is rare, accounting for approximately 5–10% of LVOT obstructive lesions (2, 3). The treatment options for LVOT lesions have been heavily debated in the literature over the last 30 years. The outcomes for both interventional and surgical options have improved but there is still no unified treatment option.

When looking at AS as a single entity, there are both surgical and interventional options for treatment. Most centers around the world use aortic balloon valvuloplasty (AoVP) as their first-line treatment. Fratz and the group from Munich in Germany reviewed their outcomes in neonates who underwent AoVP over an 18-year period. Results revealed 59% did not require surgery after 10 years. Twenty nine percent developed moderate aortic regurgitation and 19% severe regurgitation (4). The combined mortality rate was 7%. With experience and changes in technique, there was a reduction in aortic regurgitation and need for surgical intervention. The results were comparable to surgical valvotomy (4). This was further correlated by Justino and the group at Texas Children’s Hospital who found that repeat AoVP effectively treats recurrent AS and postpones the need for surgical intervention (5).

One of the limitations with AoVP is the associated vascular trauma and arterial occlusion. This procedure traditionally was performed using the femoral artery, however, carotid and axillary cutdown is now being used with more success and less complications. Rossi and the group from Porto Alegre in Brazil found rates of femoral arterial occlusion as high as 40% (6). Over a 16-year period, a clear leg length discrepancy was observed in one patient (6). Changing the access approach to carotid cutdown resulted in no carotid occlusions or vascular injuries (6). These results were corroborated by the group in Bergamo Italy, who found no significant occlusion of the right carotid artery in 29 patients for whom a carotid cutdown approach was used (7).

The alternative to AoVP is surgical valvotomy. The group at Royal Children’s Hospital in Melbourne in 2013 published their data on surgical valvotomy. A newer technique of surgical valvotomy was described with improved outcomes when compared to previous surgical data (8). There was a 64% freedom from re-intervention at 5 years and 39% at 15 years. The early mortality rate was 3% and late mortality rate was 10% with a 10-year survival rate of 88% (8). Outcomes were similar to AoVP as reported earlier (4). The valvotomy was performed through a median sternotomy followed by cardiac bypass and deep hypothermic circulatory arrest (DHCA). Longer intensive care unit stays and hospital admissions were found compared to patients who underwent AoVP (8).

For severe to critical CoA, there are both interventional and surgical options. It is well recognized that for discrete coarctation the best treatment in neonates and small infants is surgical thoracotomy with extended resection and end-to-end anastomosis (9). Wright and the group from Michigan showed that there was no need for bypass in 87% of CoA cases. There were short cross clamp times (a median time of 17 mins) and low peri-operative mortality of less than 1% (9). By performing this operation in the neonatal and early infancy period, the long-term risk of hypertension was decreased, and 1-year post operation, no patients had clinical hypertension. The need for re-intervention was also low at 6%. These results were supported by a study in 2009 by the Chicago group who found 2% early mortality and 4% re-intervention rate (10). In comparison, aortic angioplasty in infants under 3 months was found to be far inferior to surgery. There was a significantly higher rate of restenosis (up to 70% in some case series), as well as increased complications of aneurysm formation, femoral arterial occlusion, and injury (3, 11).

A major driving factor in the decision to use a hybrid approach in this case was the ability to avoid a median sternotomy and avoidance of cardiac bypass. There is accumulating evidence that infant open-heart surgery puts children at high risk of neurological and developmental disability and these deficits persist throughout childhood (12). Cardiac bypass and the use of DHCA have been associated with increased risk of seizures and worse neurological outcomes (13) DHCA has been reserved mainly for neonates and infants as it has better neurological protection and reduces metabolic demand on the developing brain (14, 15). Therefore, any avoidance of cardiac bypass theoretically should decrease this risk.

Over the last 15 years, the group from Montreal Children’s Hospital has closely followed children who have had open-heart surgery. The focus has been on the neurological and developmental outcomes of these children and some of the major risk factors and complications have been highlighted. Results have revealed that up to 40% of children with CHD undergoing cardiac bypass surgery developed abnormal neurological examinations (16). Significant microcephaly was found in 17%, and only 14% of patients had head circumference greater than the 50th percentile. Gross and fine motor delay was found in 42% of patients with 23% having global developmental delay. Behavioral issues were observed in 35% of children (16) with 30% of these children still displaying abnormalities when entering school (17). Developmental and functional testing performed at school entry showed significantly lower mean IQ scores placing them in the low average range (90–94) (12). A significant percentage of these children also exhibited a range of developmental difficulties enhancing the risk of learning challenges, low self-esteem, and decreased social participation (12).

This case series identified risk factors associated with worse neurological and developmental outcomes. A younger age at surgery, the use of DHCA, the length of DHCA, shorter duration of core cooling, and length of intensive care admission were all associated with worse long-term neurological outcomes (12, 16–18). They also noted that abnormal neurological examination post-op had higher incidence of long-term neurological and developmental abnormalities. Cardiac bypass surgery and associated post-operative complications, including low cardiac output state, thrombi from the bypass circuit, and hypoxic ischemic reperfusion injury, were hypothesized to contribute to adverse neurological outcomes (17). For this reason, avoidance of bypass surgery will likely negate these risks in the long term.

## Summary

Severe AS and CoA is a complex condition that is rarely seen. Optimal treatment has long been difficult to achieve. Using a hybrid technique by performing AoVP, surgical resection, and end-to-end anastomosis, the best treatment option for each lesion was provided. Known procedure-related complications were avoided with the use of surgical carotid cutdown. Long-term risks of neurological and developmental impairment were reduced with the avoidance of cardiac bypass and DHCA.

## Ethics Statement

The study is a retrospective case report on established, accepted practices, but combined in a new way to benefit the patient; hence, no ethical approval is required. The draft paper was sent to the patient and family. After review of the paper, the family gave consent for this publication.

## Author Contributions

Substantial contributions to the conception or design of the work: DM. Drafting the work or revising it critically for important intellectual content: DM, MC, and DT.

## Disclaimer

This article/paper/report presents independent research. The views expressed are those of the author(s) and not necessarily those of the NHS, the NIHR, or Department of Health.

## Conflict of Interest Statement

The authors declare that the research was conducted in the absence of any commercial or financial relationships that could be construed as a potential conflict of interest.

## References

[1] Williams JA Bansal AK Kim BJ Nwakanma LU Patel ND Seth AK Two thousand Blalock-Taussig shunts: a six-decade experience. Ann Thorac Surg (2007) 84(6):2070–5. 10.1016/j.athoracsur.2007.06.067 18036938

[2] Hoffman JI Kaplan S. The incidence of congenital heart disease. J Am Coll Cardiol (2002) 39:1890. 10.1016/S0735-1097(02)01886-7 12084585

[3] Tokel K Yildirim SV Varan B Ekici E. Sequential balloon dilatation for combined aortic valvular stenosis and coarctation of the aorta in a single catheterization procedure: a prognostic evaluation based on long-term follow up. J Invasive Cardiol (2006) 18(2):65–9. 16446519

[4] Fratz S Gildein HP Balling G Sebening W Genz T Eicken A Aortic valvuloplasty in pediatric patients substantially postpones the need for aortic valve surgery: a single-center experience of 188 patients after up to 17.5 years of follow-up. Circulation (2008) 117(9):1201–6. 10.1161/CIRCULATIONAHA.107.687764 18285571

[5] Petit CJ Maskatia SA Justino H Mattamal RJ Crystal MA Ing FF. Repeat balloon aortic valvuloplasty effectively delays surgical intervention in children with recurrent aortic stenosis. Catheter Cardiovasc Interv (2013) 82(4):549–55. 10.1002/ccd.24562 22815228

[6] Rossi RI Manica JL Petraco R Scott M Piazza L Machado PM. Balloon aortic valvuloplasty for congenital aortic stenosis using the femoral and the carotid artery approach: a 16-year experience from a single center. Catheter Cardiovasc Interv (2011) 78(1):84–90. 10.1002/ccd.22938 21234922

[7] Borghi A Agnoletti G Poggiani C. Surgical cutdown of the right carotid artery for aortic balloon valvuloplasty in infancy: midterm follow-up. Pediatr Cardiol (2001) 22:194. 10.1007/s002460010202 11343140

[8] Siddiqui J Brizard CP Galati JC Iyengar AJ Hutchinson D Konstantinov IE Surgical valvotomy and repair for neonatal and infant congenital aortic stenosis achieves better results than interventional catheterization. J Am Coll Cardiol (2013) 62(22):2134–40. 10.1016/j.jacc.2013.07.052 23954309

[9] Wright GE Nowak CA Goldberg CS Ohye RG Bove EL Rocchini AP. Extended resection and end-to-end anastomosis for aortic coarctation in infants: results of a tailored surgical approach. Ann Thorac Surg (2005) 80(4):1453–9. 10.1016/j.athoracsur.2005.04.002 16181886

[10] Kaushal S Backer CL Patel JN Patel SK Walker BL Weigel TJ Coarctation of the aorta: midterm outcomes of resection with extended end-to-end anastomosis. Ann Thorac Surg (2009) 88(6):1932–8. 10.1016/j.athoracsur.2009.08.035 19932265

[11] Lee CL Lin JF Hsieh KS Lin CC Huang TC. Balloon angioplasty of native coarctation and comparison of patients younger and older than 3 months. Circ J (2007) 71:1781–4. 10.1253/circj.71.1781 17965502

[12] Majnemer A Limperopoulos C Shevell M Rohlicek C Rosenblatt B Tchervenkov C. Developmental and functional outcomes at school entry in children with congenital heart defects. J Pediatr (2008) 153(1):55–60. 10.1016/j.jpeds.2007.12.019 18571536

[13] Newburger JW Jonas RA Wernovsky G Wypij D Hickey PR Kuban KC A comparison of the perioperative neurologic effects of hypothermic circulatory arrest versus low-flow cardiopulmonary bypass in infant heart surgery. N Engl J Med (1993) 329:1057–64. 10.1056/NEJM199310073291501 8371727

[14] Greeley WJ Kern FH Ungerleider RM Boyd JL III Quill T Smith LR The effect of hypothermic cardiopulmonary bypass and total circulatory arrest on cerebral metabolism in neonates, infants, and children. J Thorac Cardiovasc Surg (1991) 101:783–94. 2023435

[15] Kern FH Ungerleider RM Reves JG Quill T Smith LR Baldwin B The effects of altering pump flow rate on cerebral blood flow and metabolism in neonates, infants and children. Ann Thorac Surg (1993) 56:1366–72. 10.1016/0003-4975(93)90683-9 8267438

[16] Limperopoulos C Majnemer A Shevell MI Rohlicek C Rosenblatt B Tchervenkov C Predictors of developmental disabilities after open heart surgery in young children with congenital heart defects. J Pediatr (2002) 141(1):51–8. 10.1067/mpd.2002.125227 12091851

[17] Majnemer A Limperopoulos C Shevell MI Rohlicek C Rosenblatt B Tchervenkov C. A new look at outcomes of infants with congenital heart disease. Pediatr Neurol (2009) 40(3):197–204. 10.1016/j.pediatrneurol.2008.09.014 19218033

[18] Majnemer A Limperopoulos C Shevell M Rosenblatt B Rohlicek C Tchervenkov C. Long-term neuromotor outcome at school entry of infants with congenital heart defects requiring open-heart surgery. J Pediatr (2006) 148(1):72–7. 10.1016/j.jpeds.2005.08.036 16423601

